# Supplementary material for: New Species and Records Expand the Checklist of Cellular Slime Molds (Dictyostelids) in Jilin Province, China
Source: J Fungi (Basel). 2024 Dec 2;10(12):834. doi: 10.3390/jof10120834 (PMC11678773; doi:10.3390/jof10120834)
Supplement: Supplementary file 1 [file jof-10-00834-s001.zip › jof-3275403-supplementary.pdf]

Supplementary Table S1. A checklist of dictyostelids in Iilin province, China.

Specimen No.	Vegetation type	Substrate	Substrate No.	Strain No.	Species' name	Locality	Elevation (m)	Coordinates	References
——	broadleaf forest	soil, decayed leaves	B78001	——	*Dictyostelium mucoroides* Bref.	Jilin	————		[52]
——	forest with extensive litter layer	soil, decayed leaves	B78002	——	**Raperostelium minutum* (Raper) S. Baldauf, S. Sheikh & Thulin	Jilin	————		
——	coniferous forest	*Syringa amurensis, Pinus bungeana*	B78004	——	*Dictyostelium discoideum* Raper	Jilin	————		
——	broadleaf forest	soil, decayed leaves	B78005	——	*Polysphondylium violaceum* Bref.	Jilin	————		
MR030	forest, deciduous, broadleaf forest, deciduous pine	soil	——	——	**Heterostelium tikalense* (Vadell & Cavender) S. Baldauf, S. Sheikh & Thulin	Changchun and Wangqing	————		[51, 55]
MR031	broadleaf forest	soil	——	——	**Heterostelium candidum* (H. Hagiw.) S. Baldauf, S. Sheikh & Thulin	Changbaishan Nature Reserve	————		
HMJAU MR008	broadleaf forest	soil	——	——	*Dictyostelium culliculosum* Yu Li & Xiao L. He	Wangqing	————		[56]
HMJAU MR075	coniferous forest	soil	——	S0574-1	*Dictyostelium rosarium* Raper & Cavender	Jilin	————		[57]
HMJAU MR076	coniferous forest	soil	——	S0573-1	*Dictyostelium robustum* H. Hagiw.	Jilin	————		
HMJAU MR071	broadleaf forest	soil	——	S0109-2	**Heterostelium pseudocandidum* (H. Hagiw.) S. Baldauf, S. Sheikh & Thulin	Jilin	————		

HMJAU MR057	forest	soil	——	0082-1	**Cavenderia multistipes* (Cavender) S. Baldauf, S. Sheikh & Thulin	Hunchun	231	——	
HMJAU MR047	broadleaf forest	soil	——	0446–2	**Raperostelium gracile* (H. Hagiw.) S. Baldauf, S. Sheikh & Thulin	Jingyue, Changchun City	————		
HMJAU MR045	coniferous forest	soil	——	0385–3, 0385–4, 0385–5	*Dictyostelium clavatum* H. Hagiw.	Huangnihe, Dunhua City	————		
HMJAU MR060, MR015	grass land, coniferous forest, broadleaf forest	soil	——	0445	*Dictyostelium giganteum* B.N. Singh	Jingyue, Changchun City; Wangqing	————		[23, 51]
HMJAU MR061, MR074, MR016	coniferous forest, broadleaf forest, farmland	soil	——	0453	*Dictyostelium mucoroides* Bref.	Jingyue, Changchun City; Wangqing	————		
HMJAU MR067	——	Heropencedrymion soil	——	0320, 0337	*Polysphondylium violaceum* Bref.	Changbai Mountain Nature Reserve	————		
HMJAU MR067	coniferous forest, mixed forest	soil	——	0459, 0435, 0452, 0422 0442	*Polysphondylium violaceum* Bref.	Jingyue, Changchun City; Antu County; Jiiln	————		
HMJAU MR062	mixed forest, coniferous forest	soil	——	0080–4, 0084–4, 0085–5	*Dictyostelium recurvibasicum* Yu Li & P. Liu	Wangqing National Nature Reserve	861, 766, 761	——	
HMJAU MR055	mixed forest	soil	——	0075–4	*Dictyostelium longosporum* H. Hagiw.	Wangqing National Nature Reserve	855	——	[24, 51]
HMJAU MR064	broadleaf forest	soil	——	0073–4	**Heterostelium tenuissimum* (H. Hagiw.) S. Baldauf, S. Sheikh & Thulin	Wangqing National Nature Reserve	365	——	
HMJAU MR043	mixed forest, broadleaf forest	soil	——	0081–2, 0432-2	*Dictyostelium brefeldianum* H. Hagiw.	Wangqing National Nature Reserve, Jilin	861	——	

HMJAU MR056	broadleaf forest	soil	S0425	——	*Cavenderia parvispora* (H. Hagiw.) S. Baldauf, S. Sheikh & Thulin	Zuojia	————		
HMJAU MR058	broadleaf forest	soil	S0083-1	——	*Dictyostelium vermiforme* Vadell & Cavender	Wangqing National Nature Reserve	————		[58]
MJAU MR059	broadleaf forest	soil	S0446	——	*Dictyostelium dimigraforme* Cavender	Chanchung, Jingyuetan National Natural Park,	————		
HMJAU MR238	*Pinus koraiensis* Sieb. & Zucc. forest	soil	S4377	——	*Dictyostelium quercibrachium* Cavender, S.L. Stephenson, J.C. Landolt & Vadell	Changbai Mountain	————		[59]
HMJAU MR230	alpine birch forest	soil	S4372	——	*Dictyostelium crassicaule* H. Hagiw.	Changbai Mountain	————		
HMJAU MR303	tundra, mixed broadleaf-conifer forest	soil	5035, 5735	5035-23, 5735-3-23	*Dictyostelium mucoroides* Bref.	Changbai Mountain Nature Reserve	2038, 1720	42°03'14''09.6N, 128°03'59''52.8E, 42°04'13''76N, 128°03'56''95E	
HMJAU MR302	broadleaf forest	soil	5751, 5752	5751-1-23, 5752-3-17	*Dictyostelium discoideum* Raper	Hanconggou	776, 774	42°24'06''93N, 128°05'52''43E, 42°24'07''72N, 128°05'52''14E	
HMJAU MR305	mixed broadleaf-conifer forest	soil	5729	5729-huang-2021, 5729-bai-2021	*Dictyostelium robusticaule* Y. Li, P. Liu, Y. Zou	Xibao forestry farm	828	42°22'17''84N, 128°00'13''74E	[21]
HMJAU MR313	mixed broadleaf-conifer forest, coniferous forest	soil	5754, 5759	5754-2wang-23, 5259-1-23	*Cavenderia fasciculata* (F. Traub, H.R. Hohl & Cavender) S. Baldauf, S. Sheikh & Thulin	Hanconggou, Changbai Mountain Nature Reserve	787, 1438	42°24'11''92N, 128°05'53''34E, 42°06'25''54N, 128°05'34''13E	
HMJAU MR314	coniferous forest	soil	5759	5759-1-23, 5759-2-23	*Heterostelium pallidum* (Olive) S. Baldauf, S. Sheikh & Thulin	Changbai Mountain Nature Reserve	1,438	42°06'25''54N, 128°05'34''13E	
HMJAU MR315	mixed broadleaf-conifer forest	soil	5756	5756-1-17	*Heterostelium recretum* Y. Li, P. Liu, Y. Zou	Hanconggou	768	42°24'06''81N, 128°05'54''86E	

HMJAU MR099	broadleaf forest	soil	——	0110-1, 0113-5	*Dictyostelium microsorocarpum* Yu Li & Xiao L. He	Wangqing	——	——	[51]
HMJAU MR072	——	Soil under *Pterocarpus santalinus*	——	01020	*Heterostelium pallidum* (Olive) S. Baldauf, S. Sheikh & Thulin	Yanbian Korean Autonomous Prefecture	——	——	
HMJAU MR108	broadleaf forest	soil	——	1019	*Heterostelium filamentosum* (F. Traub, H.R. Hohl & Cavender) S. Baldauf, S. Sheikh & Thulin	Yanbian Korean Autonomous Prefecture	——	——	
HMJAU MR93045, HMJAU MR93053	secondary forest	humus	——	——	*Hagiwaraea rhizopodium* (Raper & Fennell) S. Baldauf, S. Sheikh & Thulin	Jilin	——	——	
HMJAU MR93112, HMJAU MR93129	deciduous forest	humus	——	——	*Raperostelium monochasioides* (H. Hagiw.) S. Baldauf, S. Sheikh & Thulin	Jilin	——	——	
HMJAU MR017, HMJAU MR101	mixed forest	soil, fallen leaves	——	——	*Raperostelium minutum* (Raper) S. Baldauf, S. Sheikh & Thulin	Wangqing	——	——	
HMJAU MR 430, HMJAU MR 431, HMJAU MR 432	scallion farmland	soil	6709	6709C, 6709L, 6709M	*Dictyostelium longigracilis* Z.J. Zhang, P. Liu & Y. Li sp. nov.	Changbai Korean Autonomous County	630	41°27'44''N, 127°56'31''E	This study
HMJAU MR433	broadleaf forest	soil	6718	6718S1-2B	*Dictyostelium macrosoriobrevipes* Z.J. Zhang, P. Liu & Y. Li sp. nov.	Changbai Korean Autonomous County	717.85	41°29'17''N, 127°56'32''E	
HMJAU MR 434	broadleaf forest	soil	6700	6700D	*Dictyostelium robusticaule* Y. Li, P. Liu, Y. Zou	Changbai Korean Autonomous County	651	41°28'03''N, 127°56'26''E	

HMJAU MR 435, HMJAU MR 436	broadleaf forest	soil	6718	6718-Z2, 6718-zi	*Polysphondylium patagonicum* Vadell, Cavender, Romeralo & S.L. Stephenson	Changbai Korean Autonomous County	717.85	41°29'17''N, 127°56'32''E
HMJAU MR 437	broadleaf forest	soil	6715	L1-1B	*Heterostelium candidum* (H. Hagiw.) S. Baldauf, S. Sheikh & Thulin	Changbai Korean Autonomous County	795	41°31'23''N, 127°56'58''E
HMJAU MR 438-444	scallion farmland, broadleaf forest	soil	6709, 6715, 6716, 6718, 6719	6709-2, L1-2L, L2-1A, L2113A, L2-113B, S1-3A, S2-2A	*Cavenderia aureostipes* (Cavender) S. Baldauf, S. Sheikh & Thulin	Changbai Korean Autonomous County	630, 795, 798.47, 717.85, 729.46	41°27'44''N, 127°56'31''E, 41°31'23''N, 127°56'58''E, 41°31'22''N, 127°57'0''E, 41°29'17''N, 127°56'32''E, 41°29'17''N, 127°56'34''E

* indicate the latest naming based on the new classification system [25].

References

21. Zou, Y.; Hou, J.; Guo, S.; Li, C.; Li, Z.; Stephenson, S.L.; Pavlov, I.N.; Liu, P.; Li, Y. Diversity of dictyostelid cellular slime molds, including two species new to science, in forest soils of Changbai Mountain, China. *Microbiology Spectrum* **2022**, *10*, 1–22, doi:10.1128/spectrum.02402-22.
23. Liu, P.; Li, Y. Dictyostelids from Jilin Province, China. I. *Phytotaxa* **2014**, *183*, 279–283, doi:10.11646/phytotaxa.183.4.7.
24. Liu, P.; Li, Y. Dictyostelids from Jilin Province, China II. *Phytotaxa* **2017**, *323*, 77–82, doi:10.11646/phytotaxa.323.1.6.
25. Sheikh, S.; Thulin, M.; Cavender, J.C.; Escalante, R.; Kawakami, S.-i.; Lado, C.; Landolt, J.C.; Nanjundiah, V.; Queller, D.C.; Strassmann, J.E., et al. A new classification of the dictyostelids. Protist 2018, 169, 1–28, doi:10.1016/j.protis.2017.11.001.
51. Li, Y.; Wang, Q.; Liu, P.; Zhang, B. Compilation and research on the biological diversity of Jilin Province: The volume of fungi in Gymnomycota; Jilin Education Press 2019; 10.14051/j.cnki.xdyy.2024.19.035.
52. Bai, R.L. A study on some species of Acrasiomycetes. *Acta Mycologica Sinica* **1983**, *2*, 173–178, doi:10.13346/j.mycosystema.1983.03.005.
55. He, X.L.; Li, Y. Three new records of dictyostelids in China. *Mycosystema* **2008**, *27*, 532–537.
56. He, X.-L.; Li, Y. A new species of *Dictyostelium*. *Mycotaxon* **2008**, *106*, 379–383.
57. Ren, Y.Z.; Liu, P.; Li, Y. New records of dictyostelids from China. *Nova Hedwigia* **2014**, *99*, 233–237, doi:10.1127/0029-5035/2014/0140.
58. Liu, P.; Zhang, S.; Zou, Y.; Kang, X.; Li, Y. Dictyostelids from Jilin Province, China 3: new *Cavenderia* and *Dictyostelium* records. *Mycotaxon* **2019**, *134*, 613–618, doi:10.5248/134.613.
59. Zhu, H.; Guo, S.; Xue, Q.; Li, Z.; Kang, X.; Wei, Y.; Liu, P.; Wang, Q.; Li, Y. Dictyostelids from Jilin Province, China, 4. *Mycotaxon* **2021**, *136*, 445–489, doi:10.5248/136.445.
